# Multi-Omic Candidate Screening for Markers of Severe Clinical Courses of COVID-19

**DOI:** 10.3390/jcm12196225

**Published:** 2023-09-27

**Authors:** Alexander Dutsch, Carsten Uhlig, Matthias Bock, Christian Graesser, Sven Schuchardt, Steffen Uhlig, Heribert Schunkert, Michael Joner, Stefan Holdenrieder, Katharina Lechner

**Affiliations:** 1 Department of Cardiology, German Heart Centre Munich, Technical University of Munich, Lazarettstraße 36, 80636 Munich, Germany; 2 DZHK (German Centre for Cardiovascular Research), Partner Site Munich, Munich Heart Alliance, 80336 Munich, Germany; 3 Institute for Laboratory Medicine, German Heart Centre Munich, Technical University of Munich, Lazarettstraße 36, 80636 Munich, Germany; 4 Fraunhofer Institute for Toxicology and Experimental Medicine ITEM, 30625 Hannover, Germany; 5 QuoData Gesellschaft für Qualitätsmanagement und Statistik, Fabeckstr. 43, 14195 Berlin, Germany

**Keywords:** predictive diagnostics, COVID-19 longitudinal disease course, hyperinflammation, COVID-19 coagulopathy, candidate screening, multi-omics, IL-6, D-dimers, targeted prevention, personalization

## Abstract

Background: Severe coronavirus disease 2019 (COVID-19) disease courses are characterized by immuno-inflammatory, thrombotic, and parenchymal alterations. Prediction of individual COVID-19 disease courses to guide targeted prevention remains challenging. We hypothesized that a distinct serologic signature precedes surges of IL-6/D-dimers in severely affected COVID-19 patients. Methods: We performed longitudinal plasma profiling, including proteome, metabolome, and routine biochemistry, on seven seropositive, well-phenotyped patients with severe COVID-19 referred to the Intensive Care Unit at the German Heart Center. Patient characteristics were: 65 ± 8 years, 29% female, median CRP 285 ± 127 mg/dL, IL-6 367 ± 231 ng/L, D-dimers 7 ± 10 mg/L, and NT-proBNP 2616 ± 3465 ng/L. Results: Based on time-series analyses of patient sera, a prediction model employing feature selection and dimensionality reduction through least absolute shrinkage and selection operator (LASSO) revealed a number of candidate proteins preceding hyperinflammatory immune response (denoted ΔIL-6) and COVID-19 coagulopathy (denoted ΔD-dimers) by 24–48 h. These candidates are involved in biological pathways such as oxidative stress/inflammation (e.g., IL-1alpha, IL-13, MMP9, C-C motif chemokine 23), coagulation/thrombosis/immunoadhesion (e.g., P- and E-selectin), tissue repair (e.g., hepatocyte growth factor), and growth factor response/regulatory pathways (e.g., tyrosine-protein kinase receptor UFO and low-density lipoprotein receptor (LDLR)). The latter are host- or co-receptors that promote SARS-CoV-2 entry into cells in the absence of ACE2. Conclusions: Our novel prediction model identified biological and regulatory candidate networks preceding hyperinflammation and coagulopathy, with the most promising group being the proteins that explain changes in D-dimers. These biomarkers need validation. If causal, our work may help predict disease courses and guide personalized treatment for COVID-19.

## 1. Introduction

Coronavirus disease 2019 (COVID-19), caused by severe acute respiratory syndrome coronavirus 2 (SARS-CoV-2), is a heterogeneous syndrome with varying clinical presentations and varying disease courses ranging from mild to very severe [1]. While the biological mechanisms underlying this heterogeneity are incompletely understood, the current understanding is that the host response to infection with SARS-CoV-2 is a consequence of intrinsic factors (i.e., genetic predisposition [2] and acquired risk factors [3]) as well as extrinsic factors (i.e., viral load, mode of transmission, virus variant, pre-existing infections or vaccinations) [4].

A phenotypic hallmark of severe disease courses in COVID-19 is a maladaptive, hyperinflammatory host response [5]. It is functionally characterized by elevated concentrations of pro-inflammatory cytokines such as IL-1β and IL-6, suggesting that host immune dysregulation (i.e., cytokine storm) [6] might be one crucial determinant of critical clinical courses of COVID-19 [3,5]. In that regard, severely affected COVID-19 patients had higher serum levels of IL-6, IL-7, IL-10, G-CSF, M-CSF, IP-10, MCP-1, MCP-3, MIG, and MIP-1α compared to mild cases, and higher levels of MCP-3, MIG, and MIP-1α in comparison with moderate cases [7]. Of these biomarkers, IL-6 can be routinely measured in patient serum. Elevated levels of IL-6 not only correlate with morbidity and mortality in COVID-19 patients [8] but are a druggable target. In the latter regard, the antibody tocilizumab, which targets IL-6, improved survival in hospitalized COVID-19 patients with demand for oxygen therapy and systemic inflammation. It is therefore recommended in therapy algorithms specifically for this group of patients, in addition to corticosteroids [9]. Functionally, IL-6 has been associated with increased vascular permeability and interstitial edema that worsen the respiratory situation and may play a role in respiratory failure [10,11]. Furthermore, individuals with pre-existing pro-inflammatory conditions such as type 2 diabetes mellitus (T2DM), dyslipidemia, and hypertension are at higher risk for a severe disease course of COVID-19 [12]. For example, in patients with hypertension, gene expression analyses in immune and epithelial cells, compared to non-hypertensive individuals, suggest an inflammatory predisposition caused by differential regulation of various immune cell subtypes (macrophages/inflammatory monocytes, T-cells, neutrophils) relevant to COVID-19 that is amplified in response to infection with SARS-CoV-2 [13,14].

COVID-19 was also shown to be associated with a systemic pro-thrombotic phenotype [15]. In that regard, a hallmark of COVID-19 coagulopathy is D-dimers and degradation products of D-dimers and fibrin(ogen) (FDPs). Both elevations of D-dimers and FDPs are associated with critical illness and mortality in patients with COVID-19 [16].

Of note, hyperinflammation and thrombosis (i.e., immunothrombotic dysregulation) link organ involvement and pro-thrombotic features in COVID-19 and have been suggested as markers of disease severity in COVID-19 [17]. Our group has suggested cell-free circulating nucleosome levels and citrullination as biomarkers that, among other metrics, might guide clinical triage and treatment allocation in COVID-19 patients [18].

In noncritically ill patients with COVID-19, markers for inflammation and cardiac biomarkers were predictive of post-acute COVID-19 (monocyte-to-lymphocyte ratio; NT-pro BNP) and 30-day mortality (neutrophil-to-lymphocyte ratio; NT-pro BNP) [19].

This analysis aimed to characterize an early molecular serologic fingerprint of severe COVID-19 longitudinal disease courses and, in particular, to identify a multi-omic signature preceding hyperinflammation (denoted Δ IL-6) and COVID-19 coagulopathy (denoted Δ D-dimers) in patients with severe COVID-19. As there are over 1000 potential markers in this exploratory study, but only a very limited number of datasets, the aim of this study is not to confirm potential markers as statistically significant but to identify a group of relevant candidates that could be suitable to indicate critical changes in advance of clinical deterioration.

We hypothesized that changes in IL-6 and D-dimers in severely affected COVID-19 patients are preceded by a distinct serologic signature that can be detected in patient sera 24–48 h in advance. This approach could help to predict clinical deterioration at an early stage and enable the use of preventive and personalized therapies, which are key features of predictive, personalized, and preventive (3P) medicine.

## 2. Research Design and Methods

### 2.1. Patient Inclusion and Clinical Measurements

Seven seropositive SARS-CoV-2-infected patients who were admitted to the Intensive Care Unit (ICU) of the German Heart Center Munich during the first wave of the COVID-19 pandemic in 2020 were included in this prospective analysis. Two of the seven patients died very early after admission (i.e., after one and four days). They were therefore excluded from the analysis, resulting in a dataset for the analysis of five patients. These five patients underwent longitudinal plasma profiling, including multi-omics and detailed phenotypization as well as continuous assessment of vital parameters. Blood samples were taken twice daily during the clinical routine. PCR tests for SARS-CoV-2 were performed periodically. The length of the ICU stay ranged from 11 to 29 days (median duration: 22 days). Patient characteristics were *n* = 7 and (*n* = 5); 65 ± 8 years (64 ± 9 years), 29% female (40% female), median CRP 285 ± 127 mg/dL (median CRP 300 ± 142 mg/dL), IL-6 367 ± 231 ng/L (IL-6 279 ± 187 ng/L), D-dimers 7 ± 10 mg/L (D-dimers 8.5 ± 12 mg/L), NT-proBNP 2616 ± 3465 ng/L (NT-proBNP 2425 ± 3934 ng/L) on admission. Of the five patients, three required extracorporeal membrane oxygenation (ECMO) therapy during hospitalization, none of whom survived. Two of the four patients who did not require ECMO therapy died during hospitalization. The remaining two patients showed clinical improvement and were discharged to the referring hospitals. Baseline characteristics of the entire dataset of seven patients are depicted in Table 1. Supplemental Table S1 depicts the dataset of five patients after pre-processing.

### 2.2. Plasma Measurements

Venous blood samples were drawn under standardized conditions in K3-EDTA plasma tubes (Sarstedt, Nuermbrecht, Germany), daily between 06:00 and 08:00 a.m. and a second time between 2:00 and 06:00 p.m. Samples were immediately transported to the central laboratory, centrifuged with 1600× *g* for 10 min at 20 °C (Hettich Rotina 380R), aliquoted into barcoded cryotubes, and stored at below −70 °C. Routine hematology and biochemistry testing included daily analysis of whole blood count (WBC) including hemoglobin, leukocytes platelets, and immature platelets on a Sysmex XN 2000 analyzer (Norderstedt, Germany), coagulation testing with INR, aPTT, fibrinogen, and D-dimers on a Siemens BCS XP analyzer (Erlangen, Germany), clinical chemistry analyses of potassium, sodium, calcium, creatinine, uric acid, cystatin C, creatine kinase, AST, ALT, GGT, lactate dehydrogenase, alkaline phosphatase, lipase, urea, bilirubin, protein, albumin, cholesterol, LDL-cholesterol, HDL-cholesterol, triglycerides, glucose, lactate and C-reactive protein on a Roche Cobas C 501 analyzer (Mannheim, Germany), and immunological testing including high sensitive troponin T, NT-proBNP, procalcitonin, ferritin, TSH, vitamin D, CYFRA 21-1, CA 125 and IL-6 on a Cobas E411 analyzer (Roche, Mannheim, Germany). A panel of 92 plasma proteins relevant to inflammation (Olink^®^ INFLAMMATION panel) and cardiovascular disease, including thrombosis (Olink^®^ CARDIOVASCULAR III panel), was assessed in plasma samples using a proximity extension assay by Olink© Proteomics (Uppsala, Sweden). Metabolomics analyses were done by flow injection analysis tandem mass spectrometry (FIA-MS/MS) for lipids and liquid chromatography tandem mass spectrometry (LC-MS/MS) for small molecules at the Fraunhofer Institute of Toxicology and Experimental Medicine in Hannover using the MxP Quant 500 kit (Biocrates Life Sciences AG, Innsbruck, Austria), assessing 630 metabolites from 26 biochemical classes and multiple ratios thereof.

### 2.3. Ethics

The study complied with the Declaration of Helsinki in its revised form of 2013 [20] and with Good Clinical Practice. The study was approved by the competent authorities and the Ethics Committee of the Medical Faculty at the Technical University of Munich, Germany (Number: 522/20 S-KH).

### 2.4. Statistics

This study aimed to predict the relative change in IL-6 and D-dimer biomarker levels between two consecutive days using the relative changes in candidate biomarker levels from the previous day using a linear model. The dataset included medical information from seven COVID-19 patients. After pre-processing, medical information from five patients was analyzed. Routine laboratory samples were collected frequently, while proteomic and metabolomic analyses were conducted irregularly, sometimes several times a day, resulting in 80 samples. We decided to only samples taken in the morning and pre-processed the data accordingly. The data was then divided into three groups: A (routine laboratory), B (proteomics panel), and C (metabolomics panel), measuring 84, 185, and 862 biomarkers, respectively, as shown in Supplementary Table S2. Table 2 summarizes the available samples and biomarkers before and after pre-processing.

The lower number of patient samples compared to the number of biomarkers limits the applicability of conventional methods due to the problem of multiple testing. Therefore, we developed a novel and separately published analytic method based on the LASSO-based candidate screening approach [21]. As opposed to the conventional LASSO method, this specific method does not test the specific influence of individual biomarkers but rather whether at least one of the biomarkers has a significant influence. In addition, the method provides information on the number of contributing markers. We applied this approach to three different biomarker groups (routine, proteomic, and metabolomic). We developed two models for each group, one with IL-6 as a response and one with D-dimers as a response, resulting in six models.

#### 2.4.1. Preprocessing

For each data group, pre-processing consisted of filtering by biomarker and then by sample. In the case of several samples, only the first blood sample taken in the morning was included in the final analysis. The data were then log-transformed, followed by calculating the change between two consecutive days. Finally, the data were normalized by the standard deviation and centered on the mean. Missing data points were replaced with zeros in the final stage of pre-processing for each group. This approach to treating missing data points does not significantly affect the results.

#### 2.4.2. Examining the Significance of Estimated Parameters

LASSO estimation of model parameters inherently lacks statistical significance assessment. Therefore, a new method was developed to address our research question [21]. More specifically, as separately published, we propose a test procedure based on the LASSO methodology to test the global null hypothesis of no dependence between a response variable and p predictors, where *n* observations with *n* < *p* are available [21]. The proposed procedure is similar to the F-test for a linear model, which evaluates significance based on the ratio of explained to unexplained variance [21]. However, the F-test is not suitable for models where *p* ≥ *n* [21]. This limitation is becausewhen *p* ≥ *n*, the unexplained variance is zero, and thus the F-statistic can no longer be calculated [21]. In contrast, the proposed extension of the LASSO methodology overcomes this limitation by using the number of non-zero coefficients in the LASSO model as a test statistic after suitably specifying the regularization parameter [21]. The method allows reliable analysis of high-dimensional datasets with as few as *n* = 40 observations [21]. The performance of the method was tested using a power study and was published separately [21]. In addition to the LASSO procedure, which assesses the potential for predicting levels by combining multiple biomarkers, a simple *t*-test for each individual biomarker was performed. A significance level of α = 5% was used for all tests. The significance level was corrected for multiple testing by Bonferroni correction to account for a large number of biomarkers. The software that was used is Python 3.11.0, with the main packages being scikit-learn 1.1.3 for multivariate approaches, statsmodels 0.13.5 for *t*-test statistics, and matplotlib 3.6.2 for visualization.

## 3. Results

**Figure 1:**
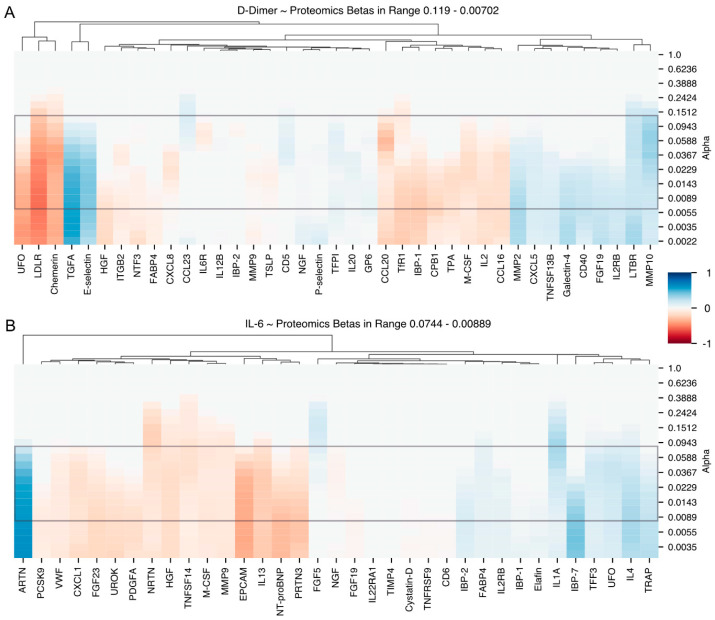
Heatmap showing (**A**) Group B explaining D-dimers and (**B**) Heatmap Group B explaining Interleukin-6. The x-axis illustrates the protein candidates selected by LASSO and the y-axis a range of alpha regularisation values determined by our novel method (as shown in Figure 2). Notably, the key alpha region for heatmap A is 0.119 to 0.00702 and for heatmap B 0.0744 to 0.00889, each marked by a grey rectangle. To facilitate the identification of potential biomarkers, candidate markers are hierarchically clustered according to their non-zero coefficient values. Colour transitions from red (negative coefficients) to white (zero) to blue (positive coefficients), with colour intensity indicating the strength of the association. Taken together, these heatmaps visualise the relationship between protein levels and two response variables: D-dimer and Interleukin-6, as explained by Group B. Abbreviations: ARTN (Artemin), CCL (CC-chemokine ligand), CD (Cluster of differentiation), CPB1 (Carboxypeptidase B1), CXCL (C-X-C motif chemokine), EPCAM (epithelial cell adhesion molecule), FABP4 (fatty acid binding protein 4), FGF (fibroblast growth factor), GPVI (Glykoprotein 6), HGF (hepatocyte growth factor), IBP (Insulin-like Growth Factor-binding Protein), IL (Interleukin), IL22RA1 (interleukin 2 receptor subunit alpha 1), IL2RB (interleukin 2 receptor subunit beta), ITGB2 (Integrin subunit beta 2), LDLR (low density lipoprotein receptor), LTBR (Lymphotxoin beta receptor), M-CSF (macrophage colony-stimulating factor), MMP (matrix metalloproteinase), NGF (nerve growth factor), NRTN (Neurturin), NTF3 (Neutrophin 3), PCSK9 (Proprotein convertase subtilisin/kexin type 9), PDGFA (Platelet-derived growth factor A), PRTN3 (Proteinase 3), TFF3 (Trefoil factor 3),TFPI (Tissue factor pathway inhibitor), TfR1 (Transferrin-recptor 1), TGFA (Transforming growth factor alpha), TIMP (Tissue inhibitor of metalloproteinase), TNFRSF (Tumor necrosis factor receptor superfamily), TPA (Plasminogen activator, tissue type), TRAP (Triiodothyronine receptor auxiliary protein), TSLP (Thymic stromal lymphopoietin), UFO (tyrosine-protein kinase receptor), UROK (Urokinase-type plasminogen activator), VWF (von Willebrand factor).

Time series analyses of patient sera revealed a number of candidates for immuno-inflammatory and thrombotic mediators, preceding a rise in D-dimers and a rise in IL-6. With our multivariate analysis pipeline, we discovered 69 potential biomarkers in the proteomics dataset, including a number of cytokines and chemokines primarily associated with immune activation and/or inflammatory responses. To decrease the candidate pool, we further reduced the number of candidate biomarkers. We were left with 48 candidate biomarkers, grouped according to their association with IL-6 and D-dimer levels. The selection criteria for the reduction to 48 candidates is described in the multivariate results section. The list can be viewed in Table 3. Finally, single relevant proteins were discussed based on a combined selection criterion that included (i) significant proteins from the multivariate analysis pipeline (Table 3) and (ii) visual selection based on the strength of the association of proteins with IL-6/D-dimers as depicted in the heatmaps (Figure 1).

### 3.1. Univariate Analysis

The results of the univariate analysis are shown in Table 4. A limited number of markers were selected based on their *t*-test values greater than 2 or 2.5 for different combinations of groups and response variables. Although no marker was found to be statistically significant under the assumption of multiple testing, it is noteworthy that IL-6 as a response variable in groups B and C produced higher *t*-values than D-dimers in these groups.

### 3.2. Multivariate Analysis

**Figure 2:**
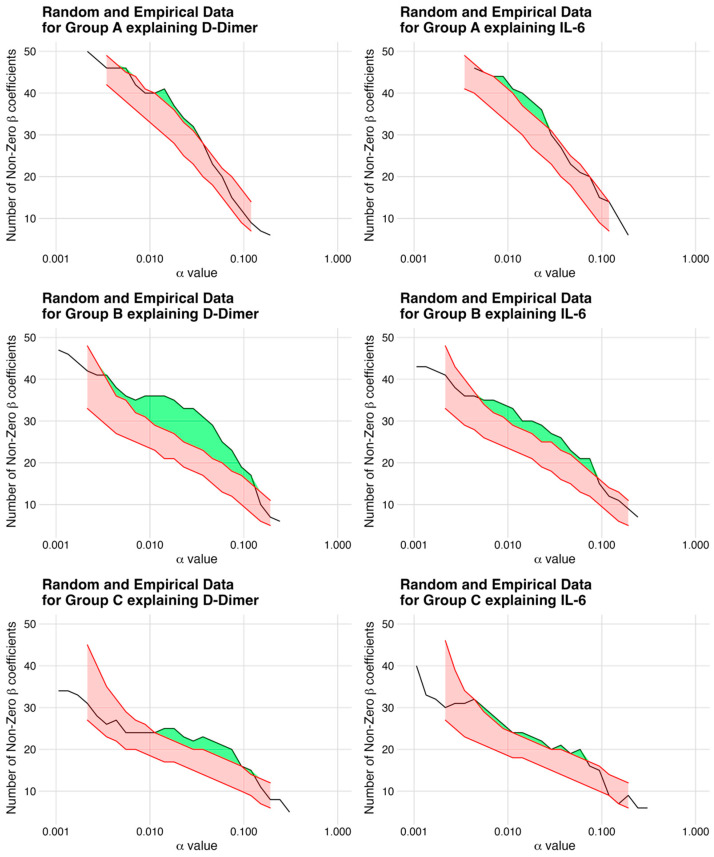
Non-Zero Coefficients in Simulated Models vs. Empirical Data. The figure depicts a plot for each model, representing the simulated data with the same correlation structure as the empirical data. The x-axis defines the regularization parameter alpha, while the y-axis shows the number of non-zero coefficients. The red bands represent the simulated data between the 5th and 95th quantile and the green band highlights the number of statistically significant candidates, which are most pronounced for group B explaining D-dimers. This plot provides a visual representation of the relevance of each model, allowing easy comparison and identification of the most informative models.

The comparison between the simulated models and the empirical data was performed using the relevance metric to identify the most informative markers, as depicted in Table 5. Of note, among the response variables, the combination of proteomics and changes in D-dimers showed the highest potential for biomarker selection, with a relevance of 28%. To generate the final list of candidate biomarkers, only the highest non-zero coefficients and markers that consistently appeared at multiple alpha values were filtered using the information from Figure 2 and the two heatmaps (Figure 1A,B). The resulting list of candidate biomarkers is presented in Table 3.

## 4. Discussion

This exploratory pilot study, using a study-specific, novel artificial intelligence algorithm [21] employing dimensionality reduction, narrowed down more than 1000 potential biomarkers to several significant blood-based biomarkers. These significant candidate biomarkers precede surges of IL-6 and D-dimers, which are prognostic markers and druggable targets of severe COVID-19 disease courses. Due to the exploratory design of this pilot study, the candidate biomarkers discussed below need validation.

### 4.1. Interleukin-6 and Hyperinflammation

Clinical deterioration in COVID-19 patients is closely linked to a dysregulated inflammatory host response to the viral infection, resulting in dysregulated cytokine release (i.e., cytokine storm) [6]. In that regard, severely affected COVID-19 patients had higher serum levels of IL-6, IL-7, IL-10, G-CSF, M-CSF, IP-10, MCP-1, MCP-3, MIG, and MIP-1α compared to mild cases and higher levels of MCP-3, MIG, and MIP-1α in comparison with moderate cases [7]. Of these biomarkers, IL-6 can be routinely measured in patient serum. Elevated levels of IL-6 correlate with morbidity and mortality in COVID-19 patients [8] and the antibody tocilizumab, which targets IL-6, improved survival in hospitalized COVID-19 patients with a demand for oxygen therapy and systemic inflammation. It is therefore recommended in therapeutic algorithms, specifically for this group of patients in addition to corticosteroid therapy [9]. Functionally, IL-6 has been associated with increased vascular permeability and interstitial edema that worsens the respiratory situation and may play a role in respiratory failure [10,11]. We therefore performed candidate screening for biomarkers in serum that predict a change in IL-6 as a surrogate for COVID-19-associated inflammatory dysregulation by applying a machine-learning approach to serial blood measurements in five severe COVID-19 disease courses. Of note, since the number of events is small relative to the number of analytes examined and because the results are in part highly correlated, a statistically validated analysis of the individual effects of these markers is not possible. For this reason, a screening was performed to determine candidate markers. We found several protein candidates associated with lung injury, neutrophil activation, and immunoinflammation/thrombosis.

#### Candidates Associated with Changes in IL-6

The physiological function of the tyrosine-protein kinase receptor UFO includes platelet activation and regulation of thrombotic responses, regulation of cell survival, cell growth, and proliferation (phosphatidylinositol-3 kinase regulatory-AKT kinase pathway), migration, and differentiation [22]. In COVID-19, Wang et al. described the tyrosine-protein kinase receptor UFO as a host-/co-receptor that promotes the entry of SARS-CoV-2 into cells—particularly in the respiratory system—to enable SARS-CoV-2 infection in the absence of ACE2 [23,24]. In line, the depletion of the tyrosine-protein kinase receptor UFO in cell lines reduced SARS-CoV-2 infection. Conversely, the overexpression in ACE2-knockout cells promoted infection, underpinning the notion that this protein may act as an alternative receptor to ACE2 [24]. We describe the tyrosine-protein kinase receptor UFO as a predictor for IL-6 rise, underpinning the notion that this protein may warrant further attention as an early predictor of hyperinflammation in COVID-19.

Interleukin-1α (IL-1α) is expressed in healthy tissue (e.g., lung epithelium) and furthermore acts as an alarmin in response to tissue damage [25]. Regarding the latter, IL-1α is released in response to tissue damage, activates macrophages (via IL-1R1), and induces IL-1β release, which in turn triggers a systemic reaction and induction of recruitment of myeloid cells to the damaged tissue. In COVID-19, IL-1α is massively released by lung epithelium cells, activating alveolar macrophages. We observed increased serum levels of IL-1α prior to a rise in IL-6 levels in the sera of severely affected COVID-19 patients, independent of ECMO therapy. This might reflect the local tissue damage and anticipate the systemic reaction, including the increase in IL-6. Overall, upregulation of IL-1α was observed to predict an inflammatory response (i.e., a rise in IL-6). Due to its known functional role and our observation of an inverse association with D-dimers, it may furthermore serve as a potential indicator of lung injury.

Artemin (Neublastin) is expressed in the central nervous system [26]. In non-COVID-19 patients, elevated levels of artemin were associated with echocardiographic markers of heart failure in patients with rheumatoid arthritis [27]. However, its function is not fully understood, and to the best of our knowledge, no literature exists on artemin in COVID-19. Our finding is thus novel and might suggest that artemin plays a role in chronic inflammatory conditions. Given the descriptive nature of our analysis, we cannot further explore its functional role in COVID-19.

We observed that IL-13 was inversely associated with IL-6. Interleukins are a functionally heterogeneous group of cytokines and may act pro-inflammatory or anti-inflammatory. IL-13 has been described as having an anti-inflammatory role by inhibiting the production of pro-inflammatory cytokines in monocytes [28]. While IL-6 reflects the TH1 response, IL-13 plays a role in the TH2 response [29]. This might provide biological plausibility for the observation that IL-13 was inversely associated with IL-6 in our study population.

Matrix metalloproteinase 9 (MMP9) and the cytokine hepatocyte growth factor (HGF) were inversely associated with both IL-6 and D-dimers and are discussed below.

### 4.2. D-Dimers and Hypercoagulopathy

More than 70% of patients who do not survive COVID-19 show evidence of thromboembolism [30]. Contrary to, e.g., coagulopathies such as disseminated intravascular coagulation (DIC), COVID-19-associated coagulopathy is typically characterized by elevated levels of D-dimers without remarkable abnormalities in other global coagulation markers [31]. This coagulopathy is associated with COVID-19 disease severity, and of note, thromboembolic complications are the most reported cause of death in COVID-19 [30,31]. We therefore performed candidate screening for biomarkers that predict changes in D-dimers as a surrogate for COVID-19-associated coagulopathy by applying a deep learning approach to serial blood measurements in five severe COVID-19 disease courses. We found several protein candidates associated with hypercoagulability, endothelial activation, platelet activation, and immunoinflammation/thrombosis. Noteworthy, aligning with physiological considerations and the prominent role of thrombosis in severe COVID-19, the combination of proteomics and change in D-dimer levels showed the highest potential for biomarker selection with a relevance of 28%.

#### Candidates Associated with Changes in D-dimers

E-selectin is expressed on endothelial cells and is a cell-surface glycol-protein that is involved in immunoadhesion [32]. SARS-CoV-2 affects vascular endothelial cells, which causes local inflammation and an imbalance between anti-coagulant and pro-thrombotic factors [31]. E-selectin is a recognized marker of endothelial activation [32]. In a single-center study of 100 hospitalized patients with COVID-19, patients requiring ICU care had higher levels of E-selectin compared with patients who did not receive ICU treatment (36.6 vs. 24.1 ng/mL; *p* < 0.001). However, E-selectin values did not differ between patients who died and survived (*p* = 0.06) nor between patients with or without a thrombotic event. [33]. In our study, E-selectin levels were positively associated with D-dimers. This is in line with a prospective study of 31 mechanically ventilated patients by Oliva et al. with COVID-19 Acute Respiratory Distress Syndrome (ARDS) and a control group of 11 patients with classical ARDS admitted to the ICU. Oliva et al. showed that at study inclusion, E-selectin levels were lower in classical ARDS than in COVID-19-related ARDS. However, levels of E-selectin did not differ in non-survivors compared to survivors. [34] Overall, the study by Oliva et al. suggests that COVID-19 ARDS is characterized by an early pulmonary endothelial injury and that E-selectin might be a marker of severe disease course and/or clinical deterioration in COVID-19 patients but does not predict survival [34]. This is in line with our findings, where E-selectin levels were found to be positively associated with levels of D-dimers. Similar results were published by Watany et al., who showed that admission levels of circulating soluble selectins P, E, and L may serve as predictors for thrombosis in COVID-19 patients and could be used to guide the decision regarding prophylactic anticoagulation [30]. In line, we observed that P-selectin levels were positively associated with levels of D-dimers. Overall, this needs to be investigated in larger cohorts to shed more light on the function of selectins in severely affected COVID-19 patients. To our knowledge, serial measurements of E-selectin in ICU patients have not been done, and our findings need to be replicated in a larger cohort of patients.

C-C motif chemokine 23 (CCL23) is a chemoattractant with chemotactic activity for monocytes, resting T-lymphocytes, and neutrophils [22]. CCL23, among the other seven proteins (IL-17C, MMP-10, FGF-19, FGF-21, FGF-23, and CXCL5), was higher in asymptomatic COVID-19 patients than in patients with symptoms [35]. This is biologically plausible since these proteins are known to be involved in tissue repair and thus may be related to the control of symptoms [35]. In non-COVID-19 stroke patients, elevated levels of CCL23 are associated with the severity of brain damage and have been suggested as possible biomarkers for assessing stroke prognosis [36]. In our study, we observed a positive association of CCL23 with D-dimers. This observation cannot be explained based on the existing literature, and further research is warranted.

The low-density lipoprotein receptor’s (LDLR) physiological function is the formation of a receptor-ligand complex and subsequent internalization of LDL particles via endocytosis [37]. It has long been known that sepsis with multiple organ failure is associated with a decrease in cholesterol levels, the latter being a predictor of mortality in sepsis [38]. In line, severe COVID-19 disease courses are associated with lower total cholesterol, lower high-density lipoprotein, and lower low-density lipoprotein (LDL) levels compared to patients with non-severe COVID-19 [39,40]. The mechanism underlying this observation might be an upregulation of LDLR expression via inhibition of the proprotein convertase subtilisin/kexin type 9 (PCSK9) by increased angiotensin II (Ang II) levels in COVID-19 patients [39]. Ang II-mediated PCSK9 inhibition and subsequent LDLR upregulation might thus be one biologically plausible pathway underlying the typical dyslipidemia associated with severe COVID-19. On the other hand, PCSK9 has a known functional role in thrombosis by promoting platelet activation, leukocyte recruitment, and clot formation through mechanisms that are unrelated to systemic lipid changes. [41] We observed an inverse association of LDLR with D-dimers [39]. Overall, in line with previous literature [38], our findings might suggest that inflammatory conditions might be linked to altered cholesterol homeostasis.

Matrix metalloproteinase 9 (MMP9) is a member of the matrix metalloproteinase (MMP) family that plays a role in the restructuring of the extracellular matrix. MMPs are involved in different physiological and pathological processes, e.g., cardiovascular diseases [42,43]. In our study, MMP9 serum levels were inversely associated with IL-6 and D-dimers. In prior studies, elevated levels of MMP9 were associated with lung tissue damage in mice overexpressing the human ACE2-receptor challenged with SARS-CoV-2. In patients with COVID-19, increased MMP9 serum levels were associated with increased mortality [44,45]. Changes in MMP9 levels may indicate lung involvement and be part of the healing process and tissue repair within the inflammatory response. Overall, alterations of MMP9 levels in advance of an inflammatory or thrombotic response depicted by a rise in D-dimers and IL-6 may be an early biomarker for these sequelae of events.

The cytokine hepatocyte growth factor (HGF) is another candidate found to be inversely regulated by IL-6 and D-dimer levels. HGF is produced by neutrophils and released upon activation of neutrophils. In that regard, HGF was found to predict disease severity (i.e., ICU admission) and mortality in COVID-19 patients [46]. On the other hand, HGF promotes tissue repair after injury (via inhibition of apoptosis of lung epithelial and endothelial cells [46], and it might also serve as a surrogate for the reparative capacity. HGF favors T_reg_ maturation, thereby acting as an anti-inflammatory protein by decreasing IL-6 and increasing IL-10. This may underlie our observation that HGF was inversely regulated with D-dimers and IL-6 and might reflect the coordinated sequelae of tissue injury and healing processes in severely affected COVID-19 patients.

Taken together, we describe candidate proteins indicative of lung injury and for regeneration and resolving inflammation that preceded changes in IL-6 and D-dimers. This molecular fingerprint might help to anticipate hallmarks of pathophysiologically relevant clinical changes (i.e., Δ IL-6 as a surrogate for systemic inflammatory response and Δ D-Dimers as a surrogate for COVID-19 coagulopathy) in COVID-19 patients and help to better understand the underlying pathophysiological sequelae. The observations from this descriptive analysis need to be validated in larger patient populations.

### 4.3. Strengths and Limitations

Our analysis has apparent limitations. First, since there are more than 1000 potential markers in this exploratory study, but at the same time only a very limited number of events, the aim of this study is not to confirm potential markers as statistically significant. Instead, the aim is to identify a group of relevant candidates that could be suitable to indicate critical changes. Our novel, study-specific biostatistical approach [21] using dimensionality reduction is an important prerequisite and motivation for further investigation by narrowing down potential candidate biomarkers. Second, this is a descriptive analysis and does not allow us to infer causality regarding disease severity and/or the extent to which the different factors contribute to the increase of IL-6 and D-dimers. Our findings thus need to be validated in larger cohorts. Third, an index that integrates these factors and takes the impact of different factors into account would be desirable. Fourth, we know the strength of the association (β-coefficient) and whether the association is positive or inverse, but we do not know the direction of the described associations. Finally, we do not have a control group to compare mild vs. severe COVID-19 or non-COVID-19 vs. COVID-19 pneumonia. It is thus unknown whether these candidate proteins play a role in mild courses of COVID-19 or in other conditions such as non-COVID-19 pneumonia.

The strengths of our study are the homogenous study population, many measuring points in the time series analysis approach applied, and, in particular, the novel mathematical model used for biostatistical analysis. [21] In the latter regard, the strengths of the biomathematical approach are several-fold; first, it makes the large data volume easier to grasp through a multi-stage process (screening—confirmation). Second, the problem of multiple testing implicit in many multi-analyte studies is avoided by the chosen statistical test approach based on the LASSO model in combination with an innovative simulation model. Thus, much better validation of the results (candidate lists) is possible. Third, by linking these candidate lists with already-known knowledge and by considering Δ IL-6 and Δ D-dimers in parallel, the understanding of further pathways can be significantly improved or simplified. Our novel approach has identified biomarkers that may help guide the prediction of clinical deterioration in patients with COVID-19. This may contribute to the application of preventive and personalized therapies.

## 5. Clinical Perspectives—Translational Outlook

Comprehensive risk stratification for COVID-19 patients remains a major challenge. The lack of biomarkers predicting deterioration might result in missed opportunities for early administration of antiviral drugs to reduce viral load. The molecular characterization of severe COVID-19 disease courses by multi-omics may contribute to a better understanding of the mechanisms involved, identify druggable targets, and ultimately contribute to ameliorated phenotypic risk stratification and treatment allocation. Traditionally, clinical research has focused on determining correlations between a limited set of variables. However, analysis of high-dimensional data, such as omics data, which poses challenges concerning data visualization and interpretation due to the large number of variables or features, requires innovative strategies. These include non-targeted analyses or phased empirical processes that sift through and hone in on the most relevant markers. In this exploratory study, there are over 1000 potential (standard and -omics) markers, but only a very limited number of datasets. Therefore, the aim of this study was not to confirm potential markers as statistically significant but to identify a group of relevant candidates that could be suitable to indicate critical changes in advance of clinical deterioration. In that regard, a central finding of our novel approach involving feature selection (syn. dimensionality reduction) [21] is that a linear model based on the group of protein biomarkers can be constructed that allows the 24 h prediction of D-dimer levels. Although the exact parameters of this model are unknown, a substantial proportion of markers from this group should likely be included in the construction of this model. Therefore, we advocate a thorough investigation of the group B proteins. Several markers within this group appear relevant in predicting D-dimer levels, as indicated in Multipanel Figure 2 and Table 5.

Overall, our work shows that (i) study-specific, novel artificial intelligence algorithms may provide independent information to conventional statistical approaches in biomedical research and that (ii) dimensionality reduction helps to transform high-dimensional data, such as omics datasets, into a lower-dimensional space while preserving relevant information.

If the associations reported prove causal, our novel approach [21] could help to predict clinical deterioration at an early stage and enable the use of preventive and personalized therapies, which are key features of 3P medicine.

## 6. Conclusions

Our novel prediction model based on time-series analysis of patient sera revealed a number of candidate proteins predicting changes in IL-6 and D-dimers as surrogates for hyperinflammatory and/or thrombotic responses in severe COVID-19 disease courses. This may contribute to a better understanding of the biological pathways involved in severe COVID-19 disease courses if these pathways prove causal. In that regard, this exploratory analysis may help guide future COVID-19 research by narrowing down the number of potential candidate markers for severe COVID-19 disease courses. We advocate that future research into biomarkers predictive of COVID-19 disease progression should focus particularly on the group of proteins that explain changes in D-dimers to guide targeted treatment.

## Figures and Tables

**Table 1:** Baseline characteristics of all patients referred to the intensive care unit of the German Heart Centre (*n* = 7).

	All Patients (*n* = 7)
Sex	
Male, *n*	5
Female, *n*	2
Age, mean (SD)	65 (8)
Days of hospitalization, mean (SD)	15 (10)
Death (if yes), *n*	5
Diabetes, *n*	3
Hypertension, *n*	5
Hypercholesterinemia, *n*	3
Smoking, *n*	0
Former Smoking, *n*	1
Coronary artery disease, *n*	2
Previous myocardial infarction, *n*	1
Previous CABG, *n*	0
Renal disease, *n*	2
Pulmonary disease, *n*	3
PCR positive, *n*	6
Intubated on admission, *n*	7
Days intubated before admission, mean (SD)	5 (4)
Intubated total (days), mean (SD)	17 (8)
Renal replacement therapy, *n*	3
Need for renal replacement therapy (days), mean (SD)	8 (11)
ECMO, *n*	3
Need for ECMO (days), mean (SD)	5
Catecholamines, *n*	6
Need for Catecholamines (days), mean (SD)	3 (3)
Antibiotics, *n*	7
Need for antibiotics (days), mean (SD)	9 (7)
Thrombotic Event, *n*	2
Haemorrhagic Event, *n*	3
Arrhythmia during hospitalization, *n*	5
Malignant Arrhythmic Event, *n*	3
Pulmonary Infiltrate, *n*	6
Medication on admission	
ASS, *n*	3
ACE-inhibitor, *n*	2
AT1-antagonist, *n*	2
Betablocker, *n*	4
Diuretics, *n*	2
Antidiabetics, *n*	3
Statin, *n*	3
Laboratory at admission	
Leukocytes (10^3^ cells/L), mean (SD)	13 (4)
Hemoglobin (g/dL), mean (SD)	10 (2)
Creatinine (mg/dL), mean (SD)	3 (2)
GFR (mL/min/1.73), mean (SD)	36 (26)
D-dimer (mg/L FEU), mean (SD)	7 (10)
Troponin high-sensitive (ng/L), mean (SD)	57 (95)
CK (U/L), mean (SD)	953 (795)
CK-MB (U/L), mean (SD)	48 (60)
CRP (mg/L), mean (SD)	285 (127)
PCT (ng/L), mean (SD)	2 (3)
IL-6 (ng/L), mean (SD)	367 (231)
NT-proBNP (ng/L), mean (SD)	2616 (3465)

Abbreviations: CABG (coronary artery bypass graft), PCR (polymerase chain reaction), ECMO (extracorporeal membrane oxygenation), ASS (aspirin; acetylsalicylic acid), ACE-inhibitor (angiotensin-converting-enzyme inhibitor), AT1-antagonist (angiotensin II receptor type 1 (AT1) antagonist), GFR (glomerular filtration rate), CK (creatine kinase), CK-MB (creatine kinase muscle brain type), CRP (C-reactive protein), PCT (procalcitonin), IL-6 (Interleukin-6), NT-proBNP (N-terminal pro–braintype natriuretic peptide).

**Table 2:** Samples and analytes. The three datasets (Routine Markers, Proteomic Markers and Metabolomic Markers) used in the study are listed, along with the number of samples and biomarkers included in each dataset. The two columns on the right (“Selected Samples and Selected Biomarkers”) represent the final processed datasets used for modeling and further analysis. The two columns on the left (“Total samples and Total Biomarkers”) show the total number of samples.

Dataset	Total Samples	Total Biomarkers	Selected Samples	Selected Biomarkers
Group A	93	84	79	48
Routine Markers				
Group B	80	185	36	184
Proteomic Markers				
Group C	80	862	26	662
Metabolomic Markers				

**Table 3:** Compiled list of potential candidates based on multivariate analysis and correlation. The table includes the results of the multivariate analysis, grouping candidates into four categories based on their beta coefficients for the corresponding response variables, D-dimers and IL-6. A positive beta coefficient greater than 0.1 is indicated by “+”, while a negative beta coefficient less than -0.1 is indicated by “−“. Proteins discussed in the Section 4 are highlighted in bold.

Group	Candidates
D-Dimer	
D-Dimer +	TGFA, CD5, MMP2, MMP10, IL2RB, **P-selectin, E-selectin**, TNR5, TNR3, CXCL5, **CCL23**, Galectin, GPVI, TN13B
D-Dimer −	**IL1α**, IBP1, FABP4, UFO, CCL16, TPA, **LDLR**, TFR1, IL6RA, IL8, CBPB1, NTF3, IL2, CCL20, Chemerin, M-CSF, **HGF, MMP9**
IL-6	
IL-6 +	IL4, FGF5, PPA5, TFF3, IBP7, **ARTN, IL1α**, IBP1, FABP4, **UFO**
Il-6 −	TNF14, UROK, PDGFA, VWF, CXCL1, EPCAM, PRTN3, **IL13**, NRTN, FGF23, M-CSF, **HGF, MMP9**

Abbreviations: ARTN (Artemin), CBPB1 (Carboxypeptidase B1), CCL (CC-chemokine ligand), CD (Cluster of differentiation), CXCL (C-X-C motif chemokine), EPCAM (epithelial cell adhesion molecule), FABP4 (fatty acid binding protein 4), FGF (fibroblast growth factor), GPVI (Glykoprotein 6), HGF (hepatocyte growth factor), IBP (Insulin-like Growth Factor-binding Protein), IL (Interleukin), IL2RB (interleukin 2 receptor subunit beta), IL6RA (interleukin 6 receptor, alpha), LDLR (low density lipoprotein receptor), M-CSF (macrophage colony-stimulating factor), MMP (matrix metalloproteinase), NRTN (Neurturin), NTF3 (Neutrophin 3), PDGFA (Platelet-derived growth factor A), PPA5 (Soluble inorganic pyrophosphatase 5), PRTN3 (Proteinase 3), TFF3 (Trefoil factor 3), TFR1 (Transferrin receptor 1), TGFA (Transforming growth factor alpha), TNF (Tumor necrosis factor), TN13B (TNF Superfamily Member 13b), TNR (Thiamine pyrophosphokinase), TPA (Plasminogen activator, tissue type), UFO (tyrosine-protein kinase receptor), UROK (Urokinase-type plasminogen activator), VWF (von Willebrand factor).

**Table 4:** Univariate analysis. A limited number of markers were selected based on their t value. The threshold for selection was set at $\left|t_{i}\right|>2$ for D-dimers group A, D-dimers group B and IL-6 group A and $\left|t_{i}\right|>2.5$ for the other three groups with i denoting the ith candidate. The t values and beta coefficients of the selected markers are shown for each corresponding group.

D-dimers				IL-6			
Marker	*t*-Value	β	Adj. *p*-Value	Marker	*t*-Value	β	Adj. *p*-Value
Group A							
C-reactive protein	2.365	0.260	1.000	Uric Acid	3.004	0.324	0.284
Mean corpuscular volume	−2.197	−0.243	1.000	Creatinine	2.596	0.284	0.892
High-sensitivity troponin T	−2.205	−0.244	1.000	Gamma-glutamyl Transferase	−2.144	−0.237	1.000
				IPC	−2.794	−0.303	0.519
				IPFAB	−2.794	−0.303	0.519
Group B							
Retinoic Acid Receptor Responder 2	−2.014	−0.326	1.000	Interleukin-1 alpha	2.799	0.433	0.302
				Fibroblast growth factor 5	2.636	0.412	0.452
				Trefoil factor 3	2.579	0.404	0.519
				Neurturin	−2.506	−0.395	0.617
				Tumor necrosis factor ligand super-family member 14	−2.962	−0.453	0.199
Group C							
Triacylglycerols (17:2_34:2)	−2.799	−0.496	0.259	Triacylglycerols (22:6_32:1)	3.855	0.618	0.020
				Triacylglycerols (16:0_37:3)	2.694	0.482	0.330
				Medium-Chain Acyl-Coenzyme A Dehydrogenase	−2.778	−0.493	0.271
				3-Hydroxy-3-Methylglutaryl-Coenzyme A Lyase	−3.241	−0.552	0.090
				Multiple Carboxylase	−3.265	−0.555	0.085

Abbreviations: CRP (C-reaktive Protein), MCV (mean corpuscular volume), hsTnT (high-sensitivity troponin T), GGT (gamma-glutamyltransferase), IPC (Immature Platelet Count), IPFAB (Immature Platelet Fraction).

**Table 5:** Multivariate Analysis. Relevance was defined as the ratio of the number of non-zero coefficients in the empirical data set to the number of non-zero coefficients above the 95th quantile of the simulation, expressed as a percentage. Absolute value represents the number of non-zero coefficients above the 95th quantile. Alpha indicates the regularisation parameter with the highest relative Relevance. Candidates refer to the number of non-zero coefficients for the model with the specified Alpha. The higher the relevance, the more markers are expected to be proportionally more relevant.

D-dimers				IL-6			
Relevance	Absolute	Alpha	Candidates	Relevance	Absolute	Alpha	Candidates
7%	3	0.0143	41	8%	3	0.0229	36
28%	8	0.0464	29	14%	3	0.0744	21
15%	3	0.0744	20	10%	2	0.0588	20

## Data Availability

The data are available on request.

## Supplementary Materials

The following supporting information can be downloaded at: https://www.mdpi.com/article/10.3390/jcm12196225/s1, Table S1: Baseline characteristics of patients from Table 1 after preprocessing (n = 5); Table S2: Combinations of datasets (shown as rows) and response variables (shown as columns) used in the study.

## References

[1] Osuchowski M.F., Winkler M.S., Skirecki T., Cajander S., Shanka-Hari M., Lachmann G., Monneret G., Venet F., Bauer M., Brunkhorst F.M. (2021). The COVID-19 puzzle: Deciphering pathophysiology and phenotypes of a new disease entity. Lancet Respir. Med..

[2] Ellinghaus D., Degenhardt F., Bujanda L., Buti M., Albillos A., Invernizzi P., Fernandez J., Prati D., Baselli G., Asselta R. (2020). Genomewide Association Study of Severe COVID-19 with Respiratory Failure. N. Engl. J. Med..

[3] Bornstein S.R., Dalan R., Hopkins D., Mingrone G., Boehm B.O. (2020). Endocrine and metabolic link to coronavirus infection. Nat. Rev. Endocrinol..

[4] Sanche S., Cassidy T., Chu P., Perelson A.S., Riberiro R.M., Ke R. (2022). A simple model of COVID-19 explains disease severity and the effect of treatments. Sci. Rep..

[5] Fajgenbaum D.C., June C.H. (2020). Cytokine Storm. N. Engl. J. Med..

[6] Huang C., Wang Y., Li X., Ren L., Zhao J., Hu Y., Zhang L., Fan G., Xu J., Gu X. (2020). Clinical features of patients infected with 2019 novel coronavirus in Wuhan, China. Lancet.

[7] Chi Y., Ge Y., Wu B., Zhan W., Wu T., Wen T., Liu J., Guo X., Huang C., Jiao Y. (2020). Serum Cytokine and Chemokine Profile in Relation to the Severity of Coronavirus Disease 2019 in China. J. Infect. Dis..

[8] Del Valle D.M., Kim-Schulze S., Huang H.-H., Beckmann N.D., Nirenberg S., Wang B., Lavin Y., Swartz T.H., Madduri D., Stock A. (2020). An inflammatory cytokine signature predicts COVID-19 severity and survival. Nat. Med..

[9] RECOVERY Collaborative Group (2021). Tocilizumab in patients admitted to hospital with COVID-19 (RECOVERY): A randomised, controlled, open-label, platform trial. Lancet.

[10] Vora S.M., Lieberman J., Wu H. (2021). Inflammasome activation at the crux of severe COVID-19. Nat. Rev. Immunol..

[11] Kaur S., Bansal R., Kollimuttathuillam S., Gowda A.M., Singh B., Mehta D., Maroules M. (2021). The looming storm: Blood and cytokines in COVID-19. Blood Rev..

[12] Gao C., Cai Y., Zhang K., Zhou L., Zhang Y., Zhang X., Li Q., Li W., Yang S., Zhao X. (2020). Association of hypertension and antihypertensive treatment with COVID-19 mortality: A retrospective observational study. Eur. Heart J..

[13] Landmesser U., Lehmann I., Eils R. (2021). Hyperinflammation as underlying mechanism predisposing patients with cardiovascular diseases for severe COVID-19. Eur. Heart J..

[14] Bartoloni E., Perricone C., Cafaro G., Gerli R. (2020). Hypertension and SARS-CoV-2 infection: Is inflammation the missing link?. Cardiovasc. Res..

[15] Levi M., Thachil J., Iba T., Levy J.H. (2020). Coagulation abnormalities and thrombosis in patients with COVID-19. Lancet Haematol..

[16] Colling M.E., Kanthi Y. (2020). COVID-19-associated coagulopathy: An exploration of mechanisms. Vasc. Med..

[17] Nicolai L., Leunig A., Brambs S., Kaiser R., Weinberger T., Weigand M., Muenchhoff M., Hellmuth J.C., Lederose S., Schulz H. (2020). Immunothrombotic Dysregulation in COVID-19 Pneumonia Is Associated with Respiratory Failure and Coagulopathy. Circulation.

[18] Cavalier E., Guiot J., Lechner K., Dutsch A., Eccleston M., Herzog M., Bygott T., Schomburg A., Kelly T., Holdenrieder S. (2021). Circulating Nucleosomes as Potential Markers to Monitor COVID-19 Disease Progression. Front. Mol. Biosci..

[19] Lionte C., Sorodoc V., Haliga R.E., Bologa C., Ceasovschih A., Petris O.R., Coman A.E., Stoica A., Sirbu O., Puha G. (2022). Inflammatory and Cardiac Biomarkers in Relation with Post-Acute COVID-19 and Mortality: What We Know after Successive Pandemic Waves. Diagnostics.

[20] Nicogossian A., Kloiber O., Stabile B. (2014). The Revised World Medical Association’s Declaration of Helsinki 2013: Enhancing the Protection of Human Research Subjects and Empowering Ethics Review Committees. World Med. Health Policy.

[21] Carsten Uhlig S.U. (2023). LASSO extension: Using the number of non-zero coefficients to test the global model hypothesis. arXiv.

[22] uniprot.org. https://www.uniprot.org.

[23] Wang S., Qiu Z., Hou Y., Deng X., Xu W., Zheng T., Wu P., Xie S., Bian W., Zhang C. (2021). AXL is a candidate receptor for SARS-CoV-2 that promotes infection of pulmonary and bronchial epithelial cells. Cell Res..

[24] Baggen J., Vanstreels E., Jansen S., Daelemans D. (2021). Cellular host factors for SARS-CoV-2 infection. Nat. Microbiol..

[25] Cavalli G., Colafrancesco S., Emmi G., Imazio M., Lopalco G., Maggio M.C., Sota J., Dinarello C.A. (2021). Interleukin 1α: A comprehensive review on the role of IL-1α in the pathogenesis and treatment of autoimmune and inflammatory diseases. Autoimmun. Rev..

[26] Baloh R.H., Tansey M.G., Lampe P.A., Fahrner T.J., Enomoto H., Simburger K.S., Leitner M.L., Araki T., Johnson E.M., Milbrandt J. (1998). Artemin, a novel member of the GDNF ligand family, supports peripheral and central neurons and signals through the GFRalpha3-RET receptor complex. Neuron.

[27] Ahlers M.J., Lowery B.D., Farber-Eger E., Wang T.J., Bradham W., Ormseth M.J., Chung C.P., Stein C.M., Gupta D.K. (2020). Heart Failure Risk Associated with Rheumatoid Arthritis-Related Chronic Inflammation. J. Am. Heart Assoc..

[28] Minty A., Chalon P., Derocq J.M., Dumon X., Guillemot J.C., Kaghad M., Labit C., Leplatois P., Liauzun P., Miloux B. (1993). Interleukin-13 is a new human lymphokine regulating inflammatory and immune responses. Nature.

[29] Marone G., Granata F., Pucino V., Pecoraro A., Heffler E., Loffredo S., Scadding G.W., Varricchi G. (2019). The Intriguing Role of Interleukin 13 in the Pathophysiology of Asthma. Frontiers in Pharmacology.

[30] Watany M.M., Abdou S., Elkolaly R., Pecoraro A., Heffler E., Loffredo S., Scadding G.W., Varricchi G. (2022). Evaluation of admission levels of P, E and L selectins as predictors for thrombosis in hospitalized COVID-19 patients. Clin Exp Med..

[31] Iba T., Connors J.M., Levy J.H. (2020). The coagulopathy, endotheliopathy, and vasculitis of COVID-19. Inflamm. Res..

[32] Silva M., Videira P.A., Sackstein R. (2018). E-Selectin Ligands in the Human Mononuclear Phagocyte System: Implications for Infection, Inflammation, and Immunotherapy. Front. Immunol..

[33] Oliva A., Rando E., Al Ismail D., De Angelis M., Cancelli F., Miele M.C., Aronica R., Mauro V., Di Timoteo F., Loffredo L. (2021). Role of Serum E-Selectin as a Biomarker of Infection Severity in Coronavirus Disease 2019. J. Clin. Med..

[34] Spadaro S., Fogagnolo A., Campo G., Zucchetti O., Verri M., Ottaviani I., Tunstall T., Grasso S., Scaramuzzo V., Murgolo F. (2021). Markers of endothelial and epithelial pulmonary injury in mechanically ventilated COVID-19 ICU patients. Crit. Care.

[35] Soares-Schanoski A., Sauerwald N., Goforth C.W., Periasamy S., Weir D.L., Lizewski S., Lizewski R., Ge Y., Kuzmina N.A., Nair V.D. (2022). Asymptomatic SARS-CoV-2 Infection Is Associated With Higher Levels of Serum IL-17C, Matrix Metalloproteinase 10 and Fibroblast Growth Factors Than Mild Symptomatic COVID-19. Front. Immunol..

[36] Simats A., García-Berrocoso T., Penalba A., Giralt D., Llovera G., Jiang Y., Ramiro L., Bustamante A., Martinez-Saez E., Canals F. (2018). CCL23: A new CC chemokine involved in human brain damage. J. Intern. Med..

[37] Goldstein J.L., Brown M.S. (1987). Regulation of low-density lipoprotein receptors: Implications for pathogenesis and therapy of hypercholesterolemia and atherosclerosis. Circulation.

[38] Fraunberger P., Schaefer S., Werdan K., Walli A.K., Seidel D. (1999). Reduction of Circulating Cholesterol and Apolipoprotein Levels during Sepsis. Clin. Chem. Lab. Med..

[39] Cure E., Cumhur Cure M. (2021). Strong relationship between cholesterol, low-density lipoprotein receptor, Na(+)/H(+) exchanger, and SARS-CoV-2: This association may be the cause of death in the patient with COVID-19. Lipids Health Dis..

[40] Sorokin A.V., Karathanasis S.K., Yang Z.H., Freeman L., Kotani K., Remaley A.T. (2020). COVID-19-Associated dyslipidemia: Implications for mechanism of impaired resolution and novel therapeutic approaches. FASEB J. Off. Publ. Fed. Am. Soc. Exp. Biol..

[41] Barale C., Melchionda E., Morotti A., Russo I. (2021). PCSK9 Biology and Its Role in Atherothrombosis. Int. J. Mol. Sci..

[42] Hopps E., Lo Presti R., Caimi G. (2017). Matrix Metalloproteases in Arterial Hypertension and their Trend after Antihypertensive Treatment. Kidney Blood Press. Res..

[43] Marchesi C., Dentali F., Nicolini E., Maresca A.M., Tayebjee M.H., Franz M., Guasti L., Venco A., Schiffrin E.L., Lip G.Y. (2012). Plasma levels of matrix metalloproteinases and their inhibitors in hypertension: A systematic review and meta-analysis. J. Hypertens..

[44] Abers M.S., Delmonte O.M., Ricotta E.E., Fintzi J., Fink D.L., de Jesus A.A.A., Zarember K.A., Alehashemi S., Oikonomou V., Desai J.V. (2021). An immune-based biomarker signature is associated with mortality in COVID-19 patients. JCI Insight.

[45] Carolina D.A.-M., Couto A.E.S., Campos L.C.B., Vasconcelos T.F., Michelon-Barbosa J., Corsi C.A.C., Mestriner F., Petroski-Moraes B.C., Garbellini-Diab M.J., Couto D.M.S. (2021). MMP-2 and MMP-9 levels in plasma are altered and associated with mortality in COVID-19 patients. Biomed. Pharmacother..

[46] Perreau M., Suffiotti M., Marques-Vidal P., Wiedemann A., Levy Y., Laouénan C., Ghosn J., Fenwick C., Comte D., Roger T. (2021). The cytokines HGF and CXCL13 predict the severity and the mortality in COVID-19 patients. Nat. Commun..

