# Supplementary material for: Multi-Omic Candidate Screening for Markers of Severe Clinical Courses of COVID-19
Source: J Clin Med. 2023 Sep 27;12(19):6225. doi: 10.3390/jcm12196225 (PMC10573369; doi:10.3390/jcm12196225)
Supplement: Supplementary file 1 [file jcm-12-06225-s001.zip › jcm-2591865-supplementary.pdf]

Supplemental Table S1. Baseline characteristics of patients from Table 1 after preprocessing (n=5). Abbreviations: CABG (coronary artery bypass graft), PCR (polymerase chain reaction), ECMO (extracorporeal membrane oxygenation), ASS (aspirin; acetylsalicylic acid), ACE-inhibitor (angiotensin-converting-enzyme inhibitor), AT1-antagonist (angiotensin II receptor type 1 (AT1) antagonist), GFR (glomerular filtration rate), CK (creatine kinase), CK-MB (creatine kinase muscle brain type), CRP (C-reactive protein), PCT (procalcitonin), IL-6 (Interleukin-6), NT-proBNP (N-terminal pro–braintype natriuretic peptide).

	**Patients (n= 5)**
Sex	
Male, n	3
Female, n	2
Age, mean (SD)	64 (9)
Days of hospitalization, mean (SD)	20 (7)
Death (if yes), n	3
Diabetes, n	3
Hypertension, n	5
Hypercholesterineamia, n	3
Smoking, n	0
Former Smoking, n	1
Coronary artery disease, n	2
Previous myocardial infarction, n	1
Previous CABG, n	0
Renal disease, n	1
Pulmonary disease, n	2
PCR positive, n	5
Intubated on admission. n	5
Days intubated before admission, mean (SD)	6 (4)
Intubated total (days), mean (SD)	22 (4)
Renal replacement therapy, n	3
Need for renal replacement therapy (days), mean (SD)	11 (11)
ECMO, n	2
Need for ECMO (days), mean (SD)	6 (9)
Catecholamines, n	5
Need for Catecholamines (days), mean (SD)	3 (4)
Antibiotics, n	5
Need for antibiotics (days), mean (SD)	13 (4)
Thrombotic Event. n	2
Haemorrhagic Event. n	2
Arrhythmia during hospitalization. n	3
Malignant Arrhythmic Event. n	1
Pulmonary Infiltrate. n	5
Medication on admission	
ASS, n	2
ACE-Inhibitor, n	2
AT1-Antagonist, n	1
Betablocker, n	3
Diuretics, n	2

Antidiabetics, n	2
Statin, n	3
Laboratory at admission	
Leukocytes (10^3 cells/L), mean (SD)	12 (4)
Hemoglobin (g/dL), mean (SD)	10 (1)
Creatinine (mg/dL), mean (SD)	3 (2)
GFR (mL/min/1,73), mean (SD)	37 (30)
D-Dimer (mg/L FEU), mean (SD)	9 (12)
Troponin (ng/L), mean (SD)	66 (114)
CK (U/L), mean (SD)	660 (506)
CK-MB (U/L), mean (SD)	53 (72)
CRP (mg/L), mean (SD)	300 (142)
PCT (ng/L), mean (SD)	2 (4)
IL-6 (ng/L), mean (SD)	279 (187)
NT-proBNP (ng/L), mean (SD)	2425 (3934)

Supplemental Table S2. Combinations of datasets (shown as rows) and response variables (shown as columns) used in the study. The datasets were divided into three groups (A, B and C) based on data availability. The response variables are D-Dimer and Interleukin 6.

**Independent variables: Biomarker candidates (relative change of level on previous day)**	**Response variable: D-Dimer (relative change of level on subsequent day)**	**Response variable: Interleukin 6 (relative change of level on subsequent day)**
Group A	Routine markers and D-Dimer	Routine markers and Interleukin 6
Group B	Proteomic markers (without D-Dimer)	Proteomic markers (without Interleukin 6)
Group C	Metabolomic markers (without D-Dimer)	Metabolomic markers (without Interleukin 6)
